# Metal-free nucleophilic trifluoromethylselenolation via an iodide-mediated umpolung reactivity of trifluoromethylselenotoluenesulfonate

**DOI:** 10.3762/bjoc.16.252

**Published:** 2020-12-10

**Authors:** Kevin Grollier, Alexis Taponard, Arnaud De Zordo-Banliat, Emmanuel Magnier, Thierry Billard

**Affiliations:** 1 Institute of Chemistry and Biochemistry (ICBMS-UMR CNRS 5246), Univ Lyon, Université Lyon 1, CNRS, CPE, INSA, 43 Bd du 11 novembre 1918, 69622 Villeurbanne, France; 2 Université Paris-Saclay, UVSQ, CNRS, UMR 8180, Institut Lavoisier de Versailles, 78035 Versailles Cedex, France; 3 CERMEP-In vivo imaging, Groupement Hospitalier Est, 59 Bd Pinel, 69677 Lyon, France

**Keywords:** fluorine, nucleophilic substitution, perfluoroalkylselenolation, selenium, trifluoromethylselenolation

## Abstract

We report herein a practical method to generate CF_3_Se^−^ (and R_F_Se^−^) anions from shelf-stable reagents under iodide activation. Metal-free nucleophilic trifluoromethylselenolations have been then performed with this in situ-generated anion. Perfluoroalkylselenolations have also been described.

## Introduction

Because of the peculiar properties of the fluorine atom, fluorinated compounds gained a growing interest over the last decades and found applications in a large panel of fields from materials to life sciences [1–15]. Fluorinated motifs bring to molecules specific and often unique electronic and physicochemical characteristics. In order to design new substrates with targeted properties, a modulation of the properties of the introduced substituents became fundamental. In this context, the development of innovative fluorinated groups recently emerged, in particular by combining heteroatoms, such as chalcogens, and fluorinated moieties [16].

Despite, the negative reputation of selenium due to its toxicity at high doses, it is an essential trace element for human physiology and biochemistry [17–20]. Furthermore, selenolated compounds found valuable applications in materials [21–23], life sciences [19–20,24–29], and drug design [30–33]. Consequently, the merging of fluorinated moieties, such as CF_3_ with selenium could constitute an interesting motif in the design of new molecules, in particular in medicinal chemistry or agrochemistry. Even if, to date, there are no CF_3_Se-containing pharmaceuticals registered [15], a recent work has demonstrated the promising development of trifluoromethylselenolated nonsteroidal anti-inflammatory drugs as potential anticancer drugs [34].

Over the last years, trifluoromethylselenolation reactions have gained a rising infatuation but, despite this recent interest, methods to introduce the CF_3_Se group into organic substrates remain limited [35–36].

**Scheme 1:**
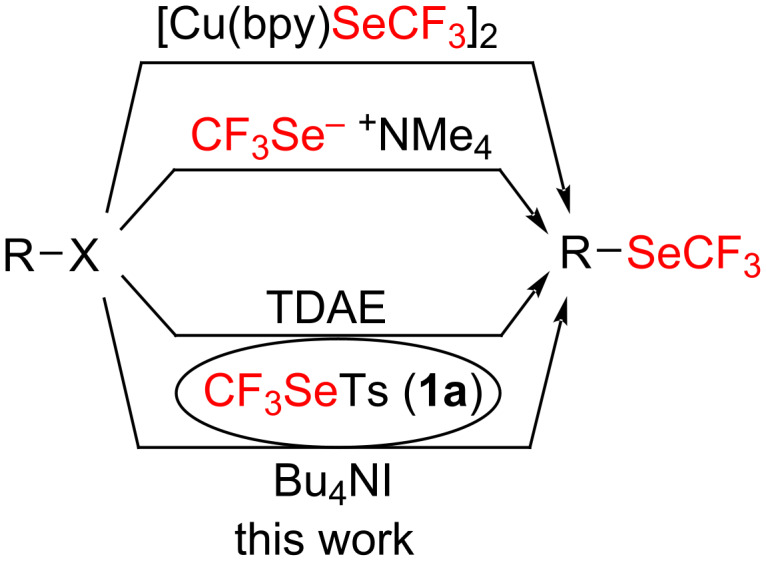
State of the art concerning the direct nucleophilic trifluoromethylselenolation.

One of the simplest ways to achieve trifluoromethylselenolated compounds is the direct nucleophilic substitution of suitable leaving groups to form the CF_3_Se–C(sp^3^) bond. This chemistry is the prerogative of the CF_3_Se^−^ anion (Scheme 1) [37–40]. However, the formation of this selenium species requires the tedious use of red elemental selenium [41] and also suffers from stability issues. To circumvent these drawbacks, a copper complex has been developed, but in this case the use of a stoichiometric amount of the metal is required [37–39].

Only a few years ago, trifluoromethylselenotoluenesulfonate (**1a**) has been developed as an efficient reagent to perform electrophilic or radical trifluoromethylselenolations [42–47]. Very recently, we demonstrated that under reductive conditions, such compounds succeeded to perform nucleophilic substitutions [48]. In this reaction, the CF_3_Se^−^ anion was in situ generated by reduction through a double electron transfer of **1a** with TDAE (tetrakis(dimethylamino)ethylene). Even though this umpolung strategy is efficient, the use of the sensitive TDAE, a strong reducing agent, could constitute a drawback for some applications. Consequently, we decided to develop a new umpolung method in non-reductive conditions.

## Results and Discussion

A few years ago, we have demonstrated that trifluoromethanesulfenamides, electrophilic trifluoromethylthiolation reagents, could also perform nucleophilic trifluoromethylthiolations through the transient formation of a CF_3_SI species which presented an inverted polarity [49–50]. Based on a similar approach, we hypothesized that the CF_3_SeI species could also possess the CF_3_Se^δ−^–I^δ+^ inverted polarity. Thus, based on the previously developed conditions, reagent **1a** was reacted with benzyl bromide (**2a**) in the presence of tetrabutylammonium iodide (TBAI) in acetone at 40 °C (Table 1, entry 1).

**Table 1:** Reaction between **1a** and **2a**.

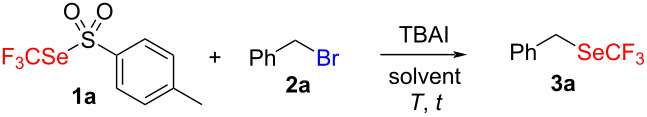							

entry	**1a** (equiv)	TBAI (equiv)	**2a** (equiv)	solvent	*T* (°C)	*t* (h)	**3a** (%)^a^

1	1	2	1	acetone	40	15	52
2	1	2	1	CH_3_CN	40	15	54
3	1	2	1	THF	40	15	61
4	1	2	1	THF	40	4	62
5	1	2	1	THF	50	4	59
6	1	2	1	THF	60	4	54
7	1	2	1	THF	25	4	47
8	1.5	3	1	THF	40	4	55
9	1	2	2	THF	40	4	89

^a^Yields determined by ^19^F NMR spectroscopy with PhOCF_3_ as an internal standard.

**Scheme 2:**
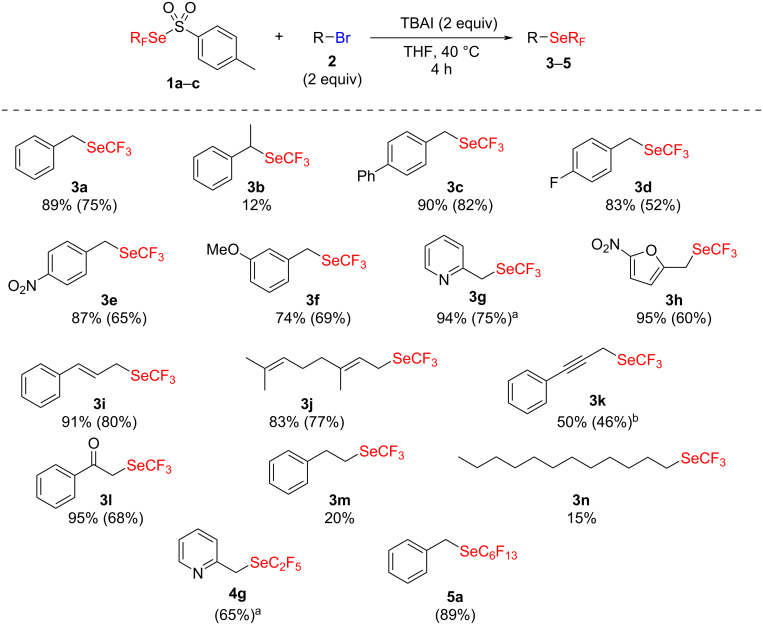
The nucleophilic fluoroalkylselenolation of alkyl bromides. Yields were determined by ^19^F NMR spectroscopy with PhOCF_3_ as an internal standard and yields of isolated products are shown in parentheses. ^a^With 1 equiv of electrophile **2**. ^b^Starting from propargyl chloride.

The observed result was moderate (Table 1, entry 1). Other solvents, which led also to satisfactory yields in the “sulfur series” were then tested. Acetonitrile did not improve the yield, however, a better one was obtained in THF (Table 1, entries 2 and 3). Interestingly, a shorter reaction time (4 h instead of 15 h) provided similar results (Table 1, entries 3 and 4). At higher temperatures, the results were not improved and even a slight decrease of the yield was observed, possibly due to an increased degradation of CF_3_SeI or CF_3_Se^−^ (Table 1, entries 5 and 6). A lower temperature also decreased the yield (Table 1, entry 7). With an excess of reagents **1a** and TBAI, leading to an excess of the CF_3_Se^−^ species, no significant improvement was observed (Table 1, entry 8). On contrary, the use of 2 equivalents of the electrophile **2a**, to ameliorate the CF_3_Se^−^ anion trapping, had a significant effect and gave a very good yield of the product (Table 1, entry 9). With the optimal conditions in hand, the reaction was exemplified with various other electrophiles (Scheme 2).

The reaction gave generally good results with reactive electrophiles such as benzylic, allylic or propargylic ones (**3a**–**k**). Noteworthy, in the reaction with 2-(bromomethyl)pyridine (**2g**) only 1 equivalent was required, maybe due to a higher reactivity. Furthermore, the reaction seems to be very sensitive to steric hindrance as illustrated by the low yield obtained for **3b**. In contrast, in the aliphatic series, only low yields were observed (**3m**,**n**) except for the activated α-bromo acetophenone (**3l**). This led us to suppose that CF_3_Se^−^ might be a poor nucleophile, which is confirmed by the medium yield observed with the propargylic substrate **3k**, where the chloride starting material was used instead of the bromide as for the other compounds. Noteworthy, because of the volatility of the obtained products, the isolated yields were sometimes significantly below the NMR yields.

Higher fluorinated homologs of **1a** were also synthesized. Consequently, an extension of this method was considered with pentafluoroethylated and tridecafluorohexylated reagents **1b** and **1c**. Good yields were obtained, in particular for **5a** which constitutes, to the best of our knowledge, the first example of a direct nucleophilic tridecafluorohexylselenolation.

**Scheme 3:**
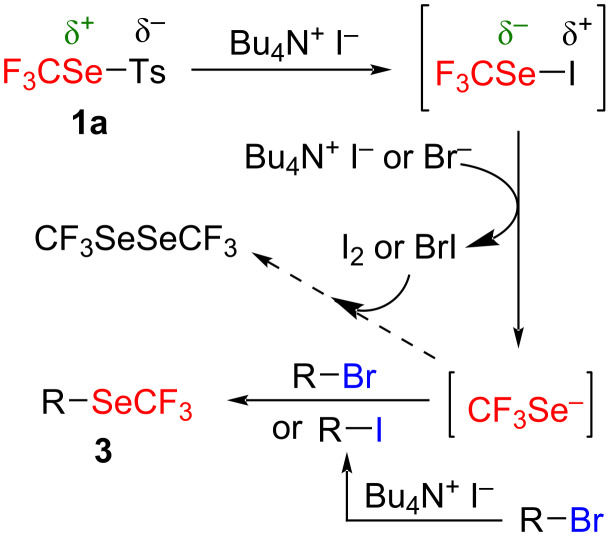
Mechanism proposal.

From a mechanistic point of view, a pathway inspired by the reaction described with trifluoromethanesulfenamides was postulated (Scheme 3) [49].

The first equivalent of iodide (from TBAI) reacts with the reagent **1a** to produce the transient species CF_3_SeI with an inverted polarity on the selenium atom. This compound then undergoes the attack of the second equivalent of iodide to generate the CF_3_Se^−^ anion with releasing of I_2_. Finally, the nucleophilic CF_3_Se^−^ can substitute the leaving group onto the electrophilic substrate **2**. However, because of the release of I_2_, as side reaction the oxidation of the CF_3_Se^−^ anion can be also envisaged. This was confirmed by the formation of 25–30% of CF_3_SeSeCF_3_ when 1 equivalent of **2a** was used (Table 1, entries 3 and 4). Consequently, the nucleophilic substitution is in competition with this relatively fast oxidation. By adding an excess of the electrophile **2**, the substitution is favored detrimentally to the oxidation. Nevertheless, with weaker or hindered electrophiles, the oxidation reaction is favored compared to the slower substitution. This is adequate with the observed results. Noteworthy, the supposed formation of I_2_ was strengthened by the appearance of a red-brown color of the reaction media which faded after the addition of sodium thiosulfate. As demonstrated in the sulfur series [49], the in situ formation of an alkyl iodide from **2**, through a Finkelstein reaction, can be also envisaged. This would not impact the reaction pathway since the released bromide can also activate the CF_3_SeI species to provide the expected CF_3_Se^−^ anion.

## Conclusion

To conclude, trifluoromethylselenotoluenesulfonate confirmed to be a versatile reagent able to perform electrophilic, radical or nucleophilic reactions depending on the conditions. The iodide-mediated, metal-free method is complementary to the previous one using TDAE. Thus, the umpolung reactivity of trifluoromethylselenotoluenesulfonate can be performed under reductive or oxidative conditions. Furthermore, this method was extended to higher fluorinated homologs allowing the first nucleophilic tridecafluorohexylselenolation.

## Experimental

Typical procedure: In a 10 mL flame-dried flask tube equipped with a magnetic stirring bar was added **1a**–**c** (0.2 mmol, 1 equiv) followed by 0.4 mL of dry THF. Then, compound **2a–n** (0.4 mmol, 2 equiv) was added followed by TBAI (0.4 mmol, 2 equiv). The tube is then sealed and the reaction mixture stirred at 40 °C for 4 h. The conversion was checked by ^19^F NMR spectroscopy with PhOCF_3_ as internal standard. After completion, the reaction mixture was partitioned between Et_2_O or pentane and water. The aqueous layer was extracted with Et_2_O and pentane and the combined organic layers were dried over MgSO_4_, filtered and concentrated to dryness. The crude residue was purified by chromatography to afford the desired products **3**, **4**, or **5**.

## Supporting Information

File 1 Additional experimental and analytical data.
