# Supplementary material for: Metal-free nucleophilic trifluoromethylselenolation via an iodide-mediated umpolung reactivity of trifluoromethylselenotoluenesulfonate
Source: Beilstein J Org Chem. 2020 Dec 10;16:3032–7. doi: 10.3762/bjoc.16.252 (PMC7736694; doi:10.3762/bjoc.16.252)
Supplement: File 1 — Additional experimental and analytical data. [file Beilstein_J_Org_Chem-16-3032-s001.pdf]

BEILSTEIN JOURNAL OF ORGANIC CHEMISTRY

Supporting Information

for

Metal-free nucleophilic trifluoromethylselenolation via an iodide-mediated umpolung reactivity of trifluoromethylselenotoluenesulfonate

Kevin Grollier, Alexis Taponard, Arnaud De Zordo-Banliat, Emmanuel Magnier and Thierry Billard

Beilstein J. Org. Chem. **2020,** *16,* 3032–3037. doi:10.3762/bjoc.16.252

Additional experimental and analytical data

License and Terms: This is a supporting information file under the terms of the Creative Commons Attribution License (https://creativecommons.org/licenses/by/4.0). Please note that the reuse, redistribution and reproduction in particular requires that the author(s) and source are credited and that individual graphics may be subject to special legal provisions.
The license is subject to the *Beilstein Journal of Organic Chemistry* terms and conditions: (https://www.beilstein-journals.org/bjoc/terms)

Table of contents

Synthesis of benzyl(trifluoromethyl)selane (**3a**) S3
Synthesis of ([1,1'-biphenyl]-4-ylmethyl)(trifluoromethyl)selane (**3c**) S3
Synthesis of (4-fluorobenzyl)(trifluoromethyl)selane **(3d**) S3
Synthesis of (4-nitrobenzyl)(trifluoromethyl)selane (**3e**) S3
Synthesis of (3-methoxybenzyl)(trifluoromethyl)selane (**3f**) S4
Synthesis of 2-(((trifluoromethyl)selanyl)methyl)pyridine (**3g**) S4
Synthesis of 2-nitro-5-(((trifluoromethyl)selanyl)methyl)furan (**3h**) S4
Synthesis of cinnamyl(trifluoromethyl)selane (**3i**) S5
Synthesis of (3,7-dimethylocta-2,6-dienyl)(trifluoromethyl)selane (**3j**) S5
Synthesis of (3-phenylprop-2-yn-1-yl)(trifluoromethyl)selane (**3k**) S5
Synthesis of 1-phenyl-2-((trifluoromethyl)selanyl)ethan-1-one (**3l**) S6
Synthesis of 2-(((1,1,2,2,2-pentafluoroethyl)selanyl)methyl)pyridine (**4g**) S6
Synthesis of benzyl(1,1,2,2,3,3,4,4,5,5,6,6,6-tridecafluorohexyl)selane (**5a**) S6
References: S7
NMR data : S8

Synthesis of benzyl(trifluoromethyl)selane **(3a)**

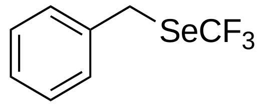

Colorless liquid
Eluent for flash chromatography: pentane 100%
^{1}H NMR (300 MHz, $CDCl_3$) δ = 7.36-7.27 (massif, 5H), 4.25 (s, 2H).
^{19}F NMR (282 MHz, $CDCl_3$) δ = -34.48 (s, 3F).
Characterization data matched that reported in the literature[1].

Synthesis of ([1,1'-biphenyl]-4-ylmethyl)(trifluoromethyl)selane **(3c)**

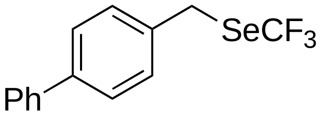

White solid
Melting point: 70°C.
Eluent for flash chromatography: pentane 100%
^{1}H NMR (300 MHz, $CDCl_3$) δ = 7.61-7.54 (massif, 4H), 7.47-7.32 (massif, 5H), 4.30 (s, 2H).
^{19}F NMR (282 MHz, $CDCl_3$) δ = -34.38 (s, 3F).
Characterization data matched that reported in the literature[2].

Synthesis of (4-fluorobenzyl)(trifluoromethyl)selane **(3d)**

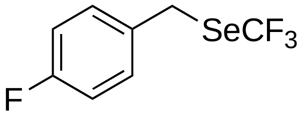

Colorless liquid
Eluent for flash chromatography: pentane 100%
^{1}H NMR (300 MHz, $CDCl_3$) δ = 7.31 (m, 2H), 7.01 (m, 2H), 4.22 (s, 2H).
^{19}F NMR (282 MHz, $CDCl_3$) δ = -34.38 (s, 3F), -114.11 (tt, J = 8.5, 5.3 Hz, 1F).
Characterization data matched that reported in the literature[3].

Synthesis of (4-nitrobenzyl)(trifluoromethyl)selane **(3e)**

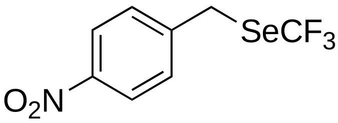

Yellow oil
Eluent for flash chromatography: pentane/Et_2O 8:2 to 7:3
^{1}H NMR (300 MHz, $CDCl_3$) δ = 8.20 (m, 2H), 7.51 (m, 2H), 4.28 (s, 2H).
^{19}F NMR (282 MHz, $CDCl_3$) δ = -34.11 (s, 3F).
Characterization data matched that reported in the literature[4].

Synthesis of (3-methoxybenzyl)(trifluoromethyl)selane **(3f)**

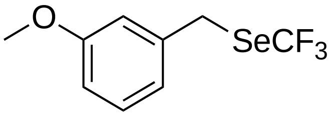

Colorless liquid
Eluent for flash chromatography: pentane/Et_2O 97:3
^{1}H NMR (300 MHz, $CDCl_3$) δ = 7.24 (t, J = 8.0 Hz, 1H), 6.93 (m, 1H), 6.88 (t, J = 2.3 Hz, 1H), 6.82 (ddd, J = 7.9, 2.2, 0.9 Hz, 1H), 4.22 (s, 2H), 3.81 (s, 3H).
^{19}F NMR (282 MHz, $CDCl_3$) δ = - 34.50 (s, 3F).
Characterization data matched that reported in the literature[4].

Synthesis of 2-(((trifluoromethyl)selanyl)methyl)pyridine **(3g)**

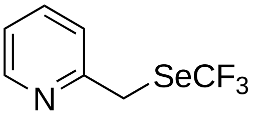

Yellowish oil
Only 1 eq. of alkyl halide was used instead of 2 eq.
Saturated aqueous $NaHCO_3$ was used instead of water during work-up
Eluent for flash chromatography: pentane/Et_2O 8:2 to 7:3
^{1}H NMR (300 MHz, $CDCl_3$) δ = 8.55 (d, J = 4.9 Hz, 1H), 7.67 (td, J = 7.7, 1.8 Hz, 1H), 7.33 (d, J = 7.8 Hz, 1H), 7.20 (ddd, J = 7.6, 4.9, 1.1 Hz, 1H), 4.37 (s, 2H).
^{19}F NMR (282 MHz, $CDCl_3$) δ = -34.58 (s, 3F).
Characterization data matched that reported in the literature[5].

Synthesis of 2-nitro-5-(((trifluoromethyl)selanyl)methyl)furan **(3h)**

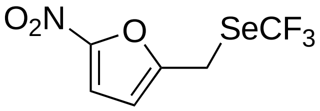

Brownish oil
Eluent for flash chromatography: pentane/Et_2O 8:2
^{1}H NMR (300 MHz, $CDCl_3$) δ = 7.27 (d, J = 3.7 Hz, 1H), 6.53 (d, J = 3.7 Hz, 1H), 4.20 (s, 2H).
^{19}F NMR (282 MHz, $CDCl_3$) δ = -34.43 (s, 3F).
Characterization data matched that reported in the literature[5].

Synthesis of cinnamyl(trifluoromethyl)selane **(3i)**

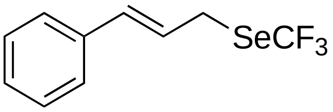

Colorless liquid
Eluent for flash chromatography: pentane 100%
^{1}H NMR (300 MHz, $CDCl_3$) δ = 7.39-7.23 (massif, 5H), 6.58 (d, J = 15.6 Hz, 1H), 6.33 (dt, J = 15.5, 7.7 Hz, 1H), 3.84 (d, J = 7.7 Hz, 2H).
^{19}F NMR (282 MHz, $CDCl_3$) δ = -33.80 (s, 3F).
Characterization data matched that reported in the literature[4].

Synthesis of (3,7-dimethylocta-2,6-dienyl)(trifluoromethyl)selane **(3j)**

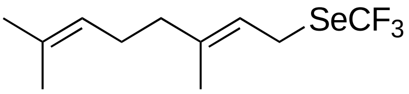

Colorless liquid
Eluent for flash chromatography: pentane 100%
^{1}H NMR (300 MHz, $CDCl_3$) δ = 5.37 (t, J = 8.4 Hz, 1H), 5.08-5.03 (m, 1H), 3.69 (d, J = 8.3 Hz, 2H), 2.14-2.00 (massif, 4H), 1.69 (s, 3H), 1.68 (s, 3H), 1.60 (s, 3H).
^{19}F NMR (282 MHz, $CDCl_3$) δ = -34.03 (s, 3F).
Characterization data matched that reported in the literature[6].

Synthesis of (3-phenylprop-2-yn-1-yl)(trifluoromethyl)selane **(3k)**

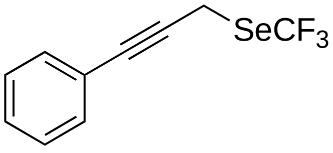

Colorless liquid
Eluent for flash chromatography: pentane 100%
^{1}H NMR (300 MHz, $CDCl_3$) δ = 7.42 (m, 2H), 7.35-7.29 (massif, 4H), 3.91 (s, 2H).
^{19}F NMR (282 MHz, $CDCl_3$) δ = -34.61 (s, 3F).
Characterization data matched that reported in the literature[4].

Synthesis of 1-phenyl-2-((trifluoromethyl)selanyl)ethan-1-one **(3l)**

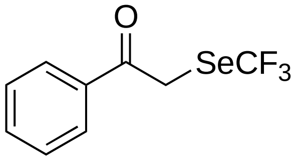

Yellow oil
Eluent for flash chromatography: pentane/Et_2O 95:5
^{1}H NMR (300 MHz, $CDCl_3$) δ = 7.97 (m, 2H), 7.63 (t, J = 7.4, 1.2 Hz, 1H), 7.51 (m, 2H), 4.63 (s, 2H).
^{19}F NMR (282 MHz, $CDCl_3$) δ = -34.19 (s, 3F).
Characterization data matched that reported in the literature[4].

Synthesis of 2-(((1,1,2,2,2-pentafluoroethyl)selanyl)methyl)pyridine **(4g)**

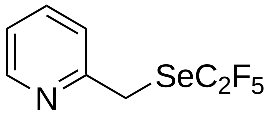

Yellowish oil
Only 1 equiv of the alkyl halide was used instead of 2 equiv.
A saturated aqueous $NaHCO_3$ was used instead of water during work-up
Eluent for flash chromatography: pentane/Et_2O 8:2 to 7:3
^{1}H NMR (300 MHz, $CDCl_3$) δ = 8.55 (ddd, J = 4.9, 1.8, 0.9 Hz, 1H), 7.67 (td, J = 7.7, 1.8 Hz, 1H), 7.33 (d, J = 7.9 Hz, 1H), 7.20 (ddd, J = 7.6, 5.0, 1.2 Hz, 1H), 4.39 (s, 2H).
^{19}F NMR (282 MHz, $CDCl_3$) δ = -83.62 (t, J = 4.0 Hz, 3F), -91.92 (q, J = 4.2 Hz, 2F).
Characterization data matched that reported in the literature[5].

Synthesis of benzyl(1,1,2,2,3,3,4,4,5,5,6,6,6-tridecafluorohexyl)selane **(5a)**

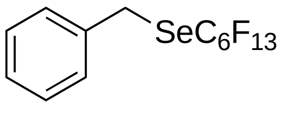

Yellowish oil
Eluent for flash chromatography: pentane 100%
^{1}H NMR (300 MHz, $CDCl_3$) δ = 7.37-7.27 (massif, 5H), 4.29 (s, 2H).
^{19}F NMR (282 MHz, $CDCl_3$) δ = -80.76 (tt, J = 10.0, 2.5 Hz, 3F), -86.73 (ddt, J = 17.5, 10,5, 3.5 Hz, 2F), -118.27 (m, 2F), -121.44 (m, 2F), -122.75 (m, 2F), -126.08 (m, 2F).
Characterization data matched that reported in the literature[7].

References:

1. Glenadel, Q.; Ismalaj, E.; Billard, T. *J. Org. Chem.* **2016,** *81*, 8268-8275.
2. Chen, X.-L.; Zhou, S.-H.; Lin, J.-H.; Deng, Q.-H.; Xiao, J.-C. *Chem. Commun.* **2019,** *55*, 1410-1413.
3. Ghiazza, C.; Khrouz, L.; Billard, T.; Monnereau, C.; Tlili, A. *Eur. J. Org. Chem.* **2020,** *2020*, 1559-1566.
4. Dong, T.; He, J.; Li, Z.-H.; Zhang, C.-P. *ACS Sustainable Chem. Eng.* **2018,** *6*, 1327-1335.
5. Ghiazza, C.; Kataria, A.; Tlili, A.; Toulgoat, F.; Billard, T. *Asian J. Org. Chem.* **2019,** *8*, 675-678.
6. Rong, M.; Huang, R.; You, Y.; Weng, Z. *Tetrahedron* **2014,** *70*, 8872-8878.
7. Glenadel, Q.; Ismalaj, E.; Billard, T. *Eur. J. Org. Chem.* **2017,** *2017*, 530-533.

NMR data :

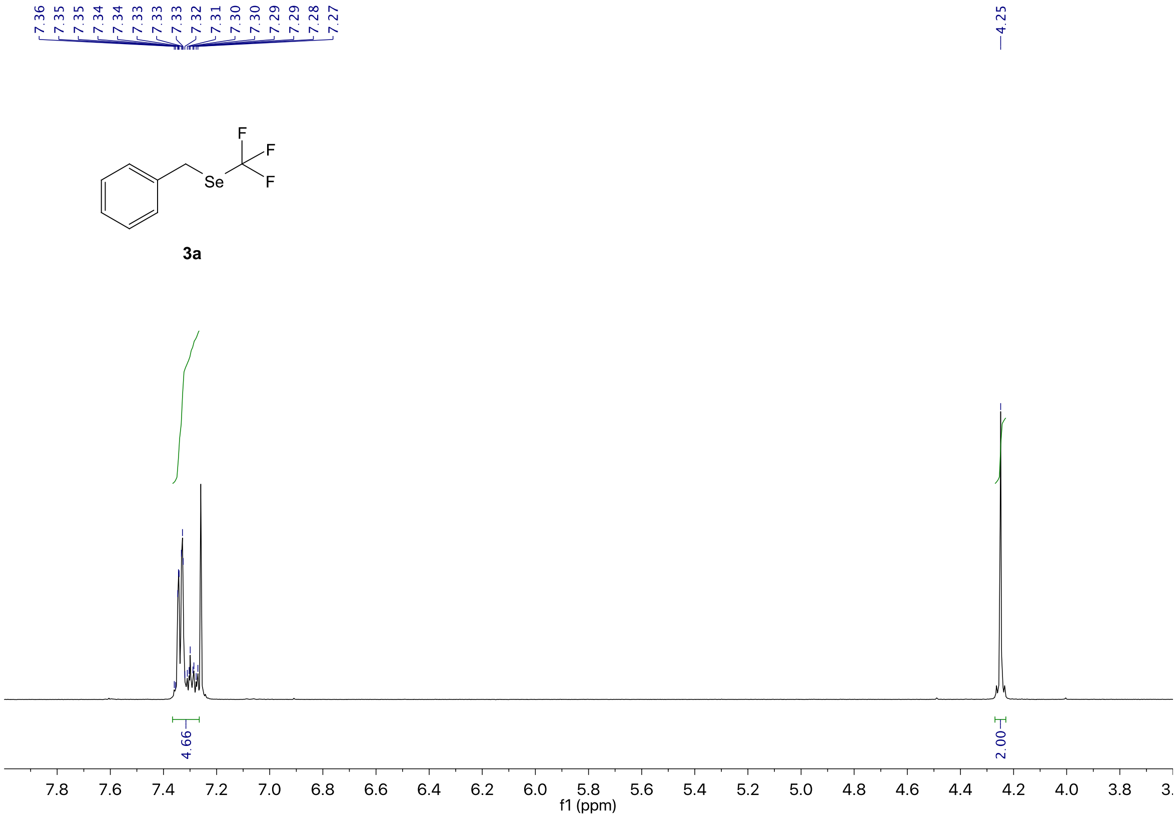
7.36
7.35
7.35
7.34
7.34
7.33
7.33
7.33
7.32
7.31
7.30
7.30
7.29
7.29
7.28
7.27
4.25
Se
F
F
F
3a
4.66
2.00
f1 (ppm)

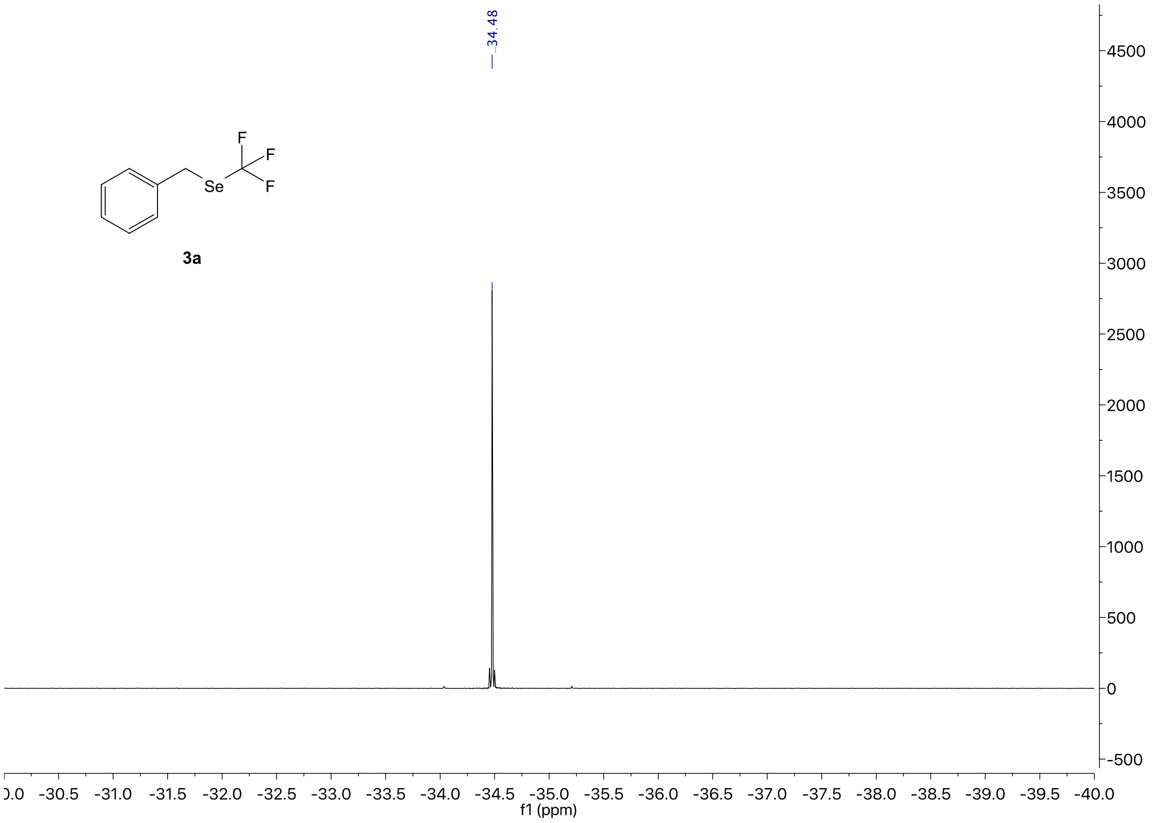
-34.48
Se
F
F
F
3a
f1 (ppm)

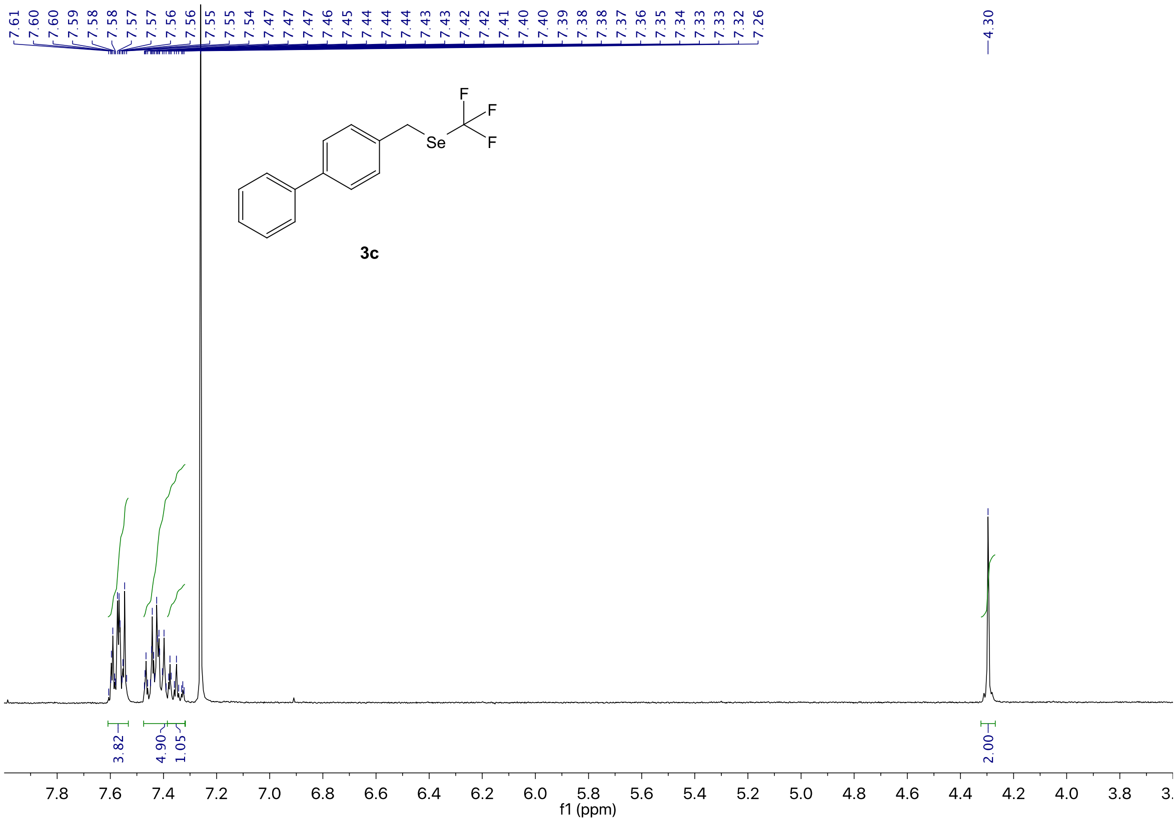
3c
f1 (ppm)

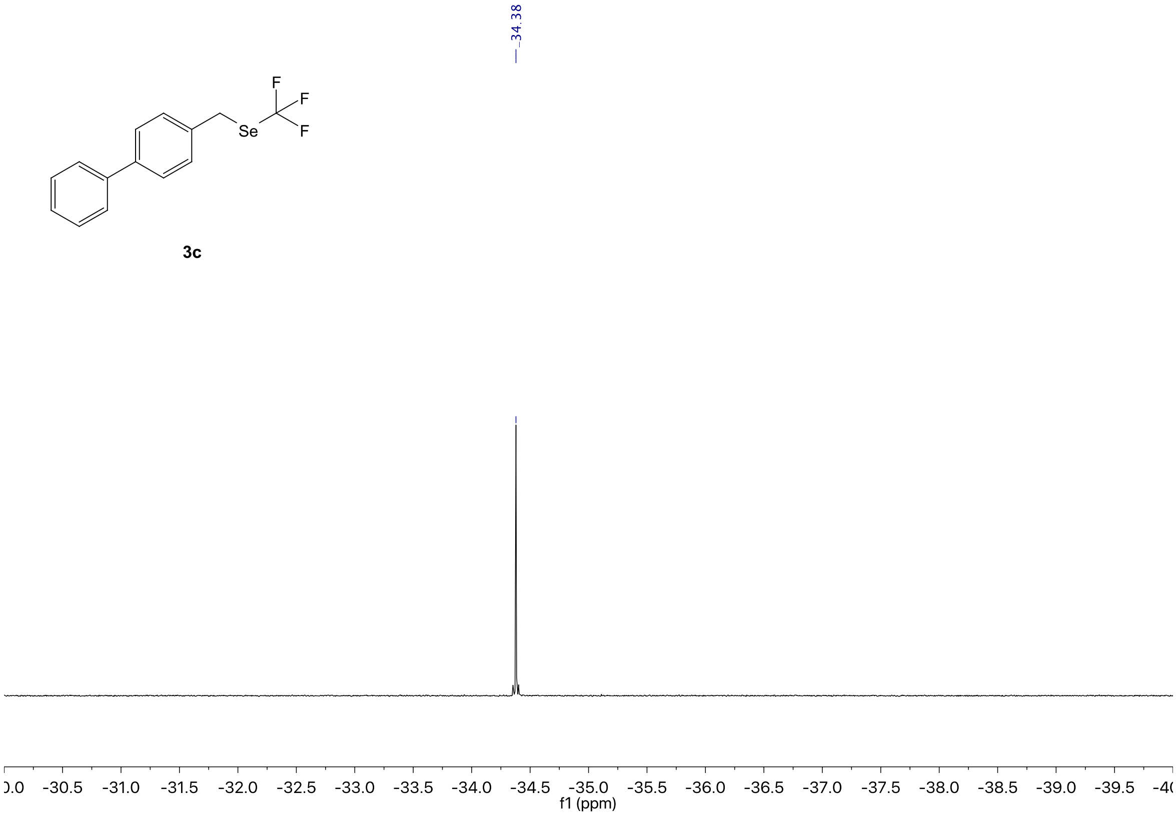
3c
f1 (ppm)

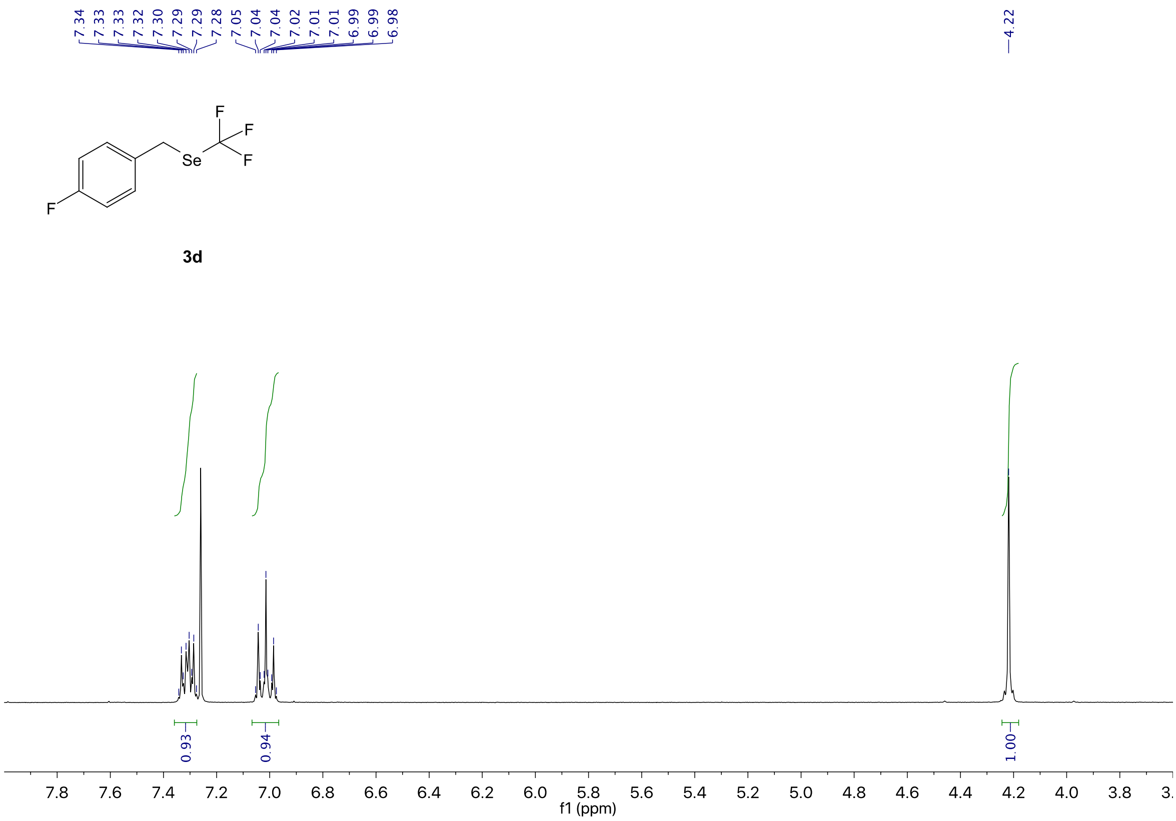
7.34
7.33
7.33
7.32
7.30
7.29
7.29
7.28
7.05
7.04
7.04
7.02
7.01
7.01
6.99
6.99
6.98
4.22
F
F
F
Se
F
0.93
0.94
1.00
7.8
7.6
7.4
7.2
7.0
6.8
6.6
6.4
6.2
6.0
5.8
5.6
5.4
5.2
5.0
4.8
4.6
4.4
4.2
4.0
3.8
f1 (ppm)

3d

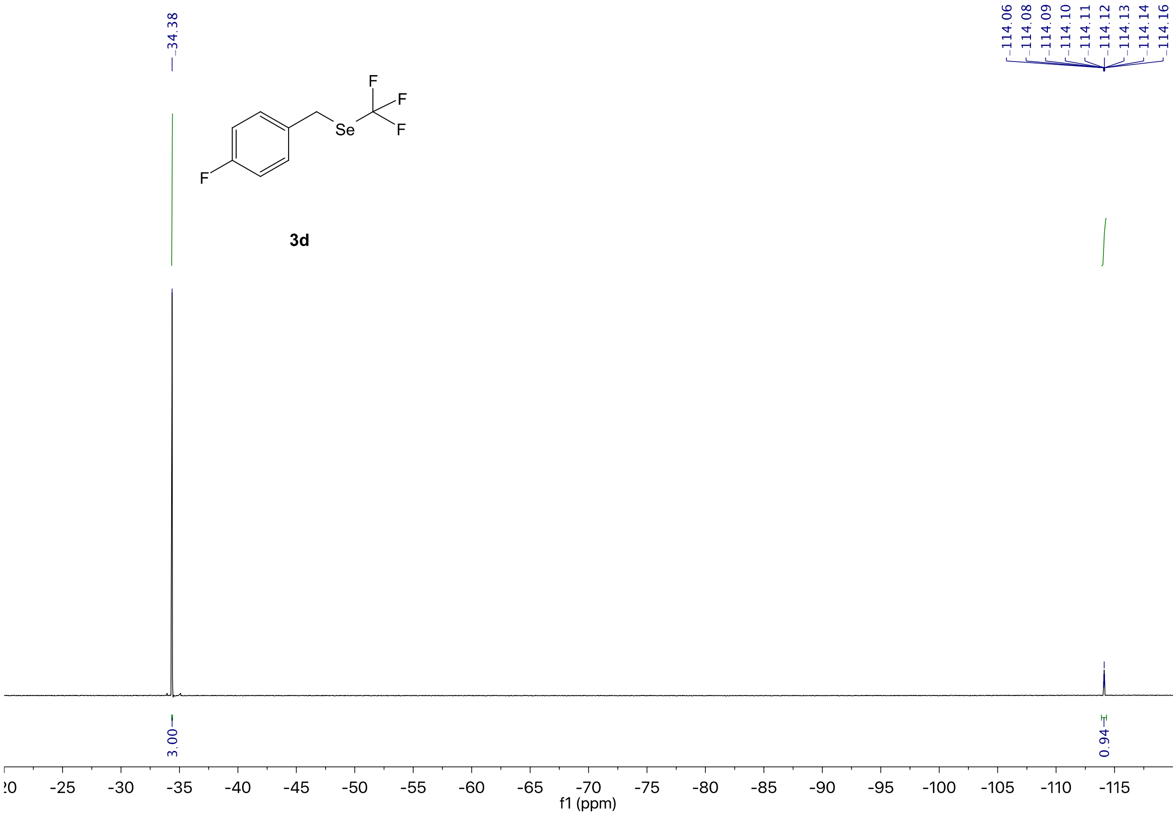
-34.38
-114.06
-114.08
-114.09
-114.10
-114.11
-114.12
-114.13
-114.14
-114.16
F
F
F
Se
F
3.00
0.94
-25
-30
-35
-40
-45
-50
-55
-60
-65
-70
-75
-80
-85
-90
-95
-100
-105
-110
-115
f1 (ppm)

3d

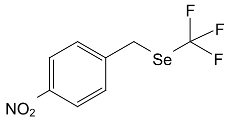
F
F
F
Se
NO2

3e

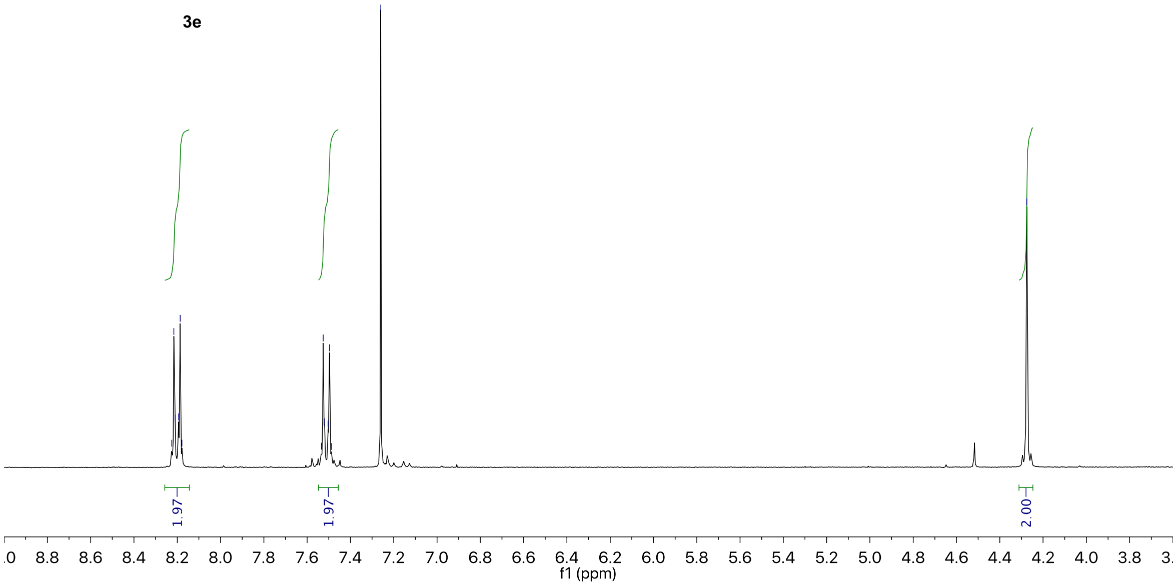
8.22
8.22
8.21
8.19
8.19
8.18
7.53
7.53
7.52
7.50
7.50
7.49
7.26
4.28
1.97
1.97
2.00
f1 (ppm)

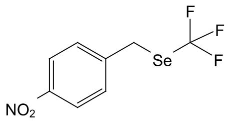
F
F
F
Se
NO2

3e

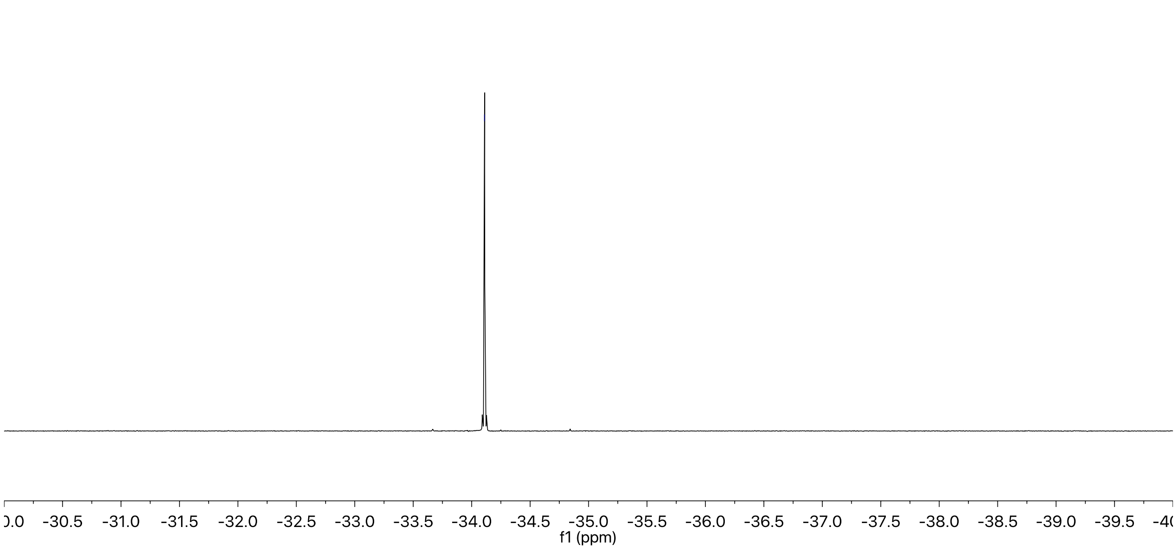
-34.11
f1 (ppm)

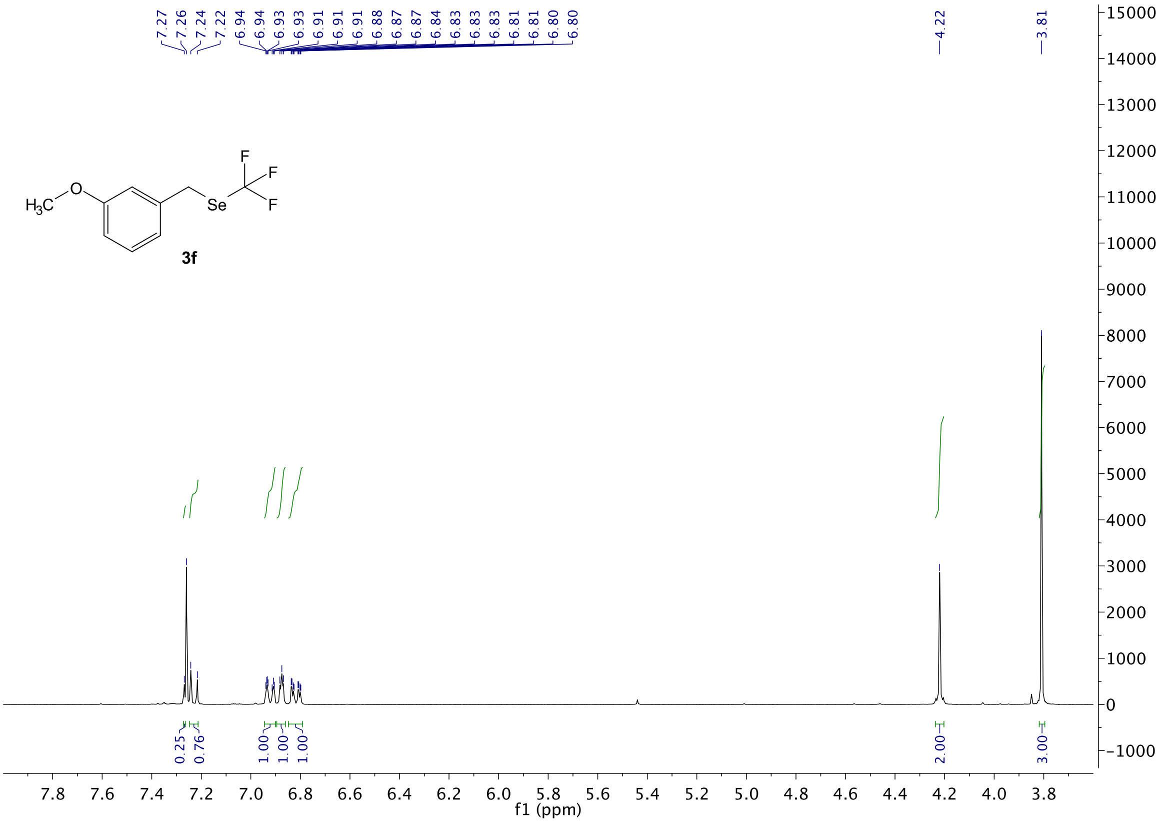
3f
f1 (ppm)

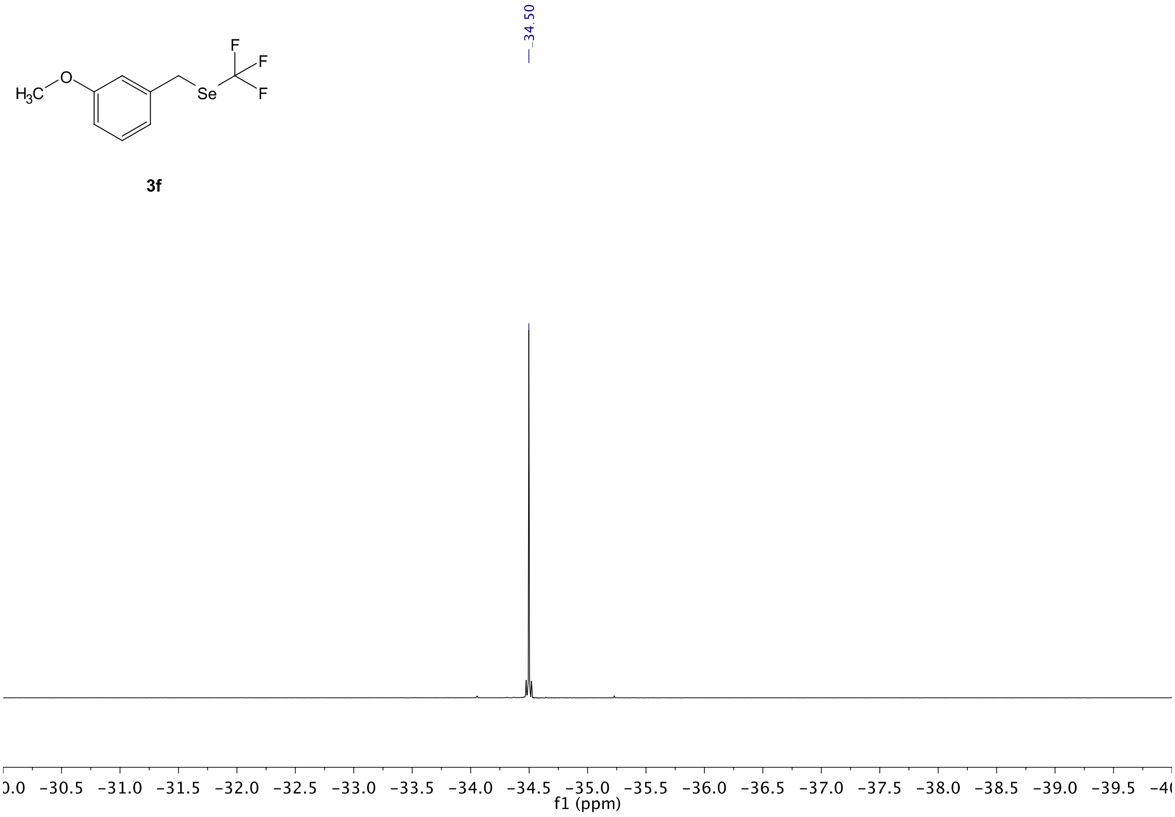
3f
f1 (ppm)

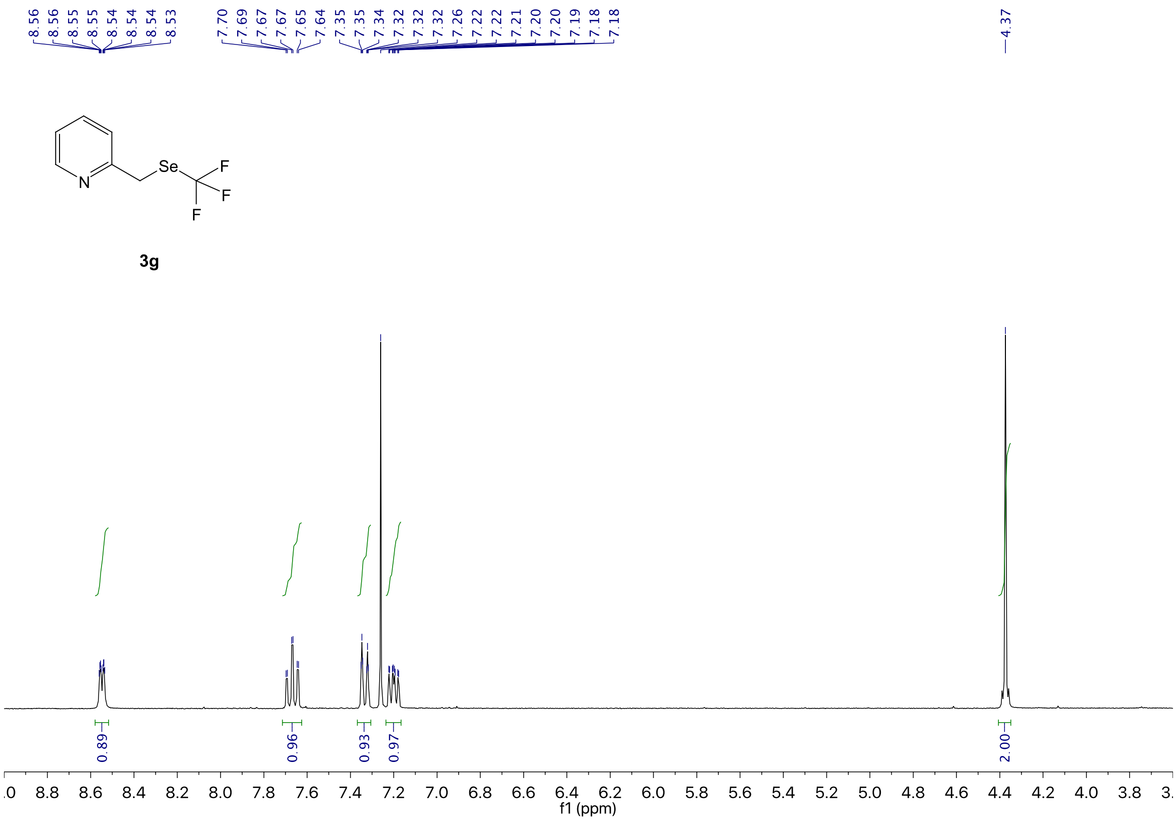
8.56 8.56 8.55 8.55 8.54 8.54 8.54 8.53
7.70 7.69 7.67 7.67 7.65 7.64 7.35 7.35 7.34 7.32 7.32 7.26 7.22 7.22 7.21 7.20 7.20 7.19 7.18 7.18
4.37
Se
F
F
F
N
3g
0.89
0.96
0.93
0.97
2.00
f1 (ppm)

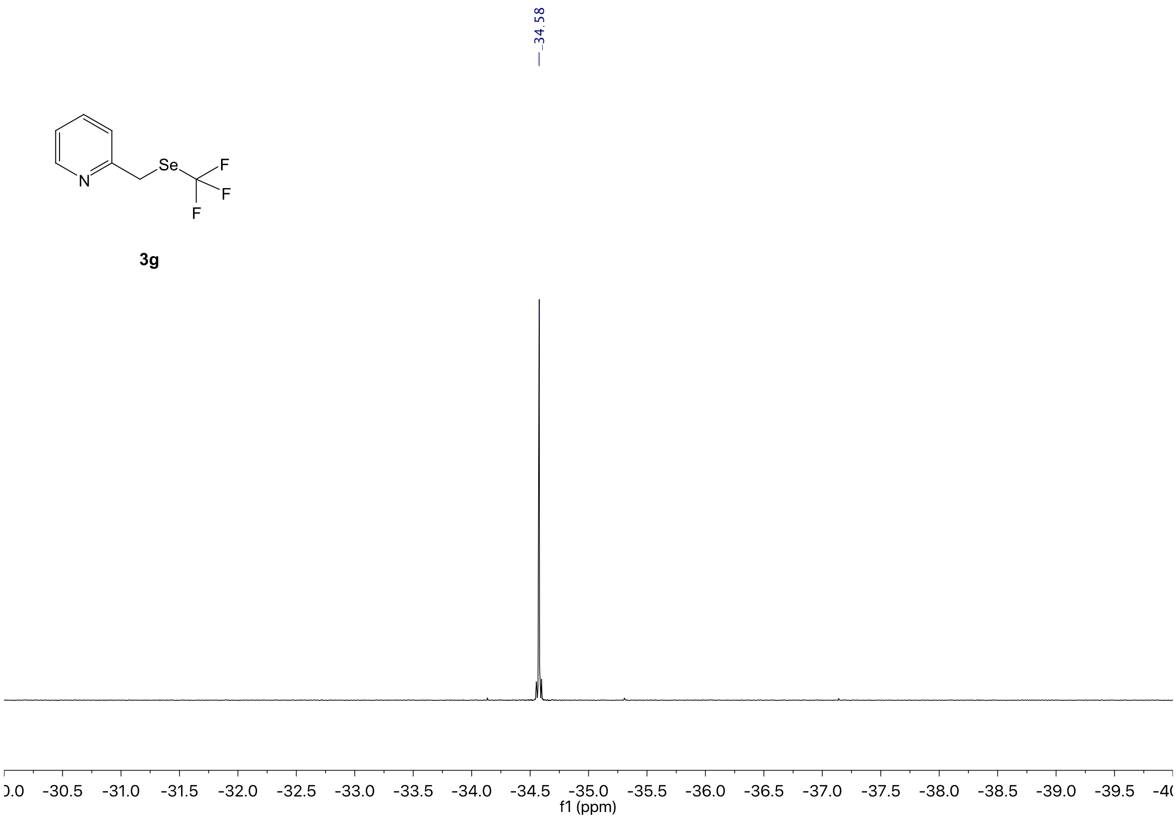
-34.58
Se
F
F
F
N
3g
f1 (ppm)

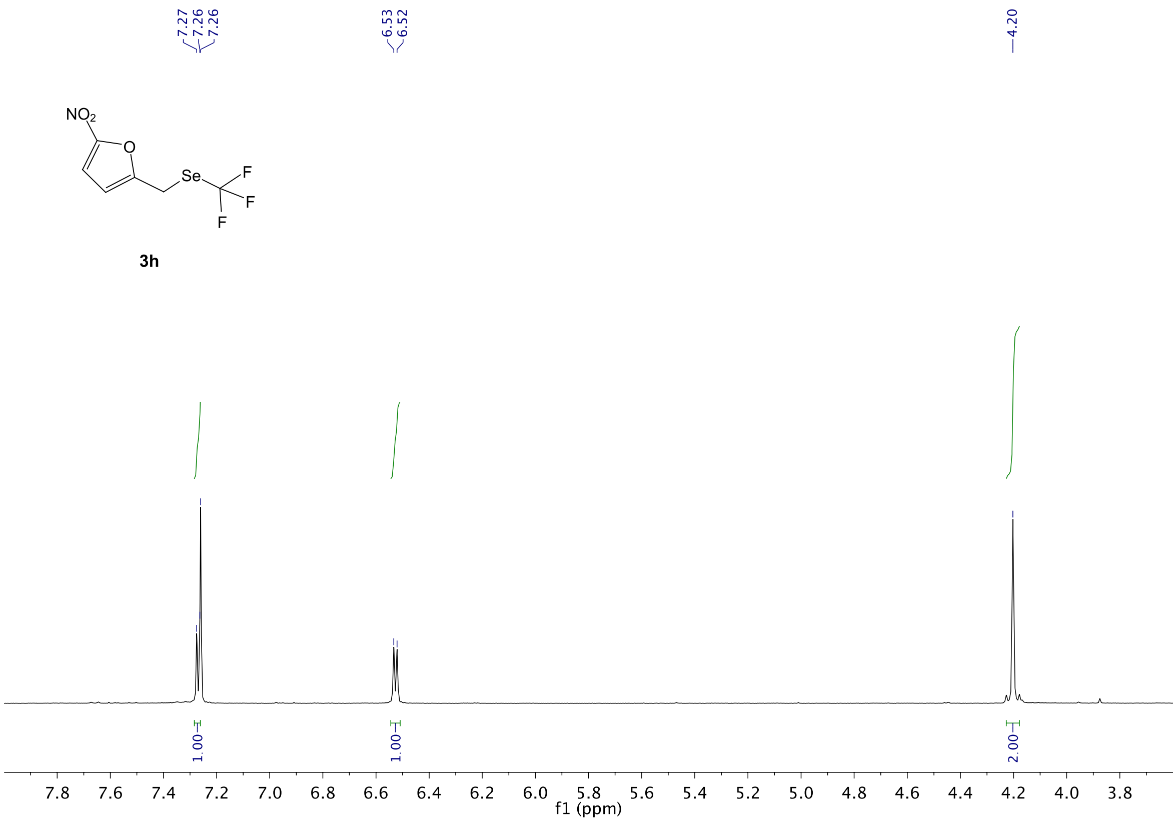

7.27
7.26
7.26
6.53
6.52
4.20
NO2
O
Se
F
F
F
1.00
1.00
2.00
7.8 7.6 7.4 7.2 7.0 6.8 6.6 6.4 6.2 6.0 5.8 5.6 5.4 5.2 5.0 4.8 4.6 4.4 4.2 4.0 3.8
f1 (ppm)

3h

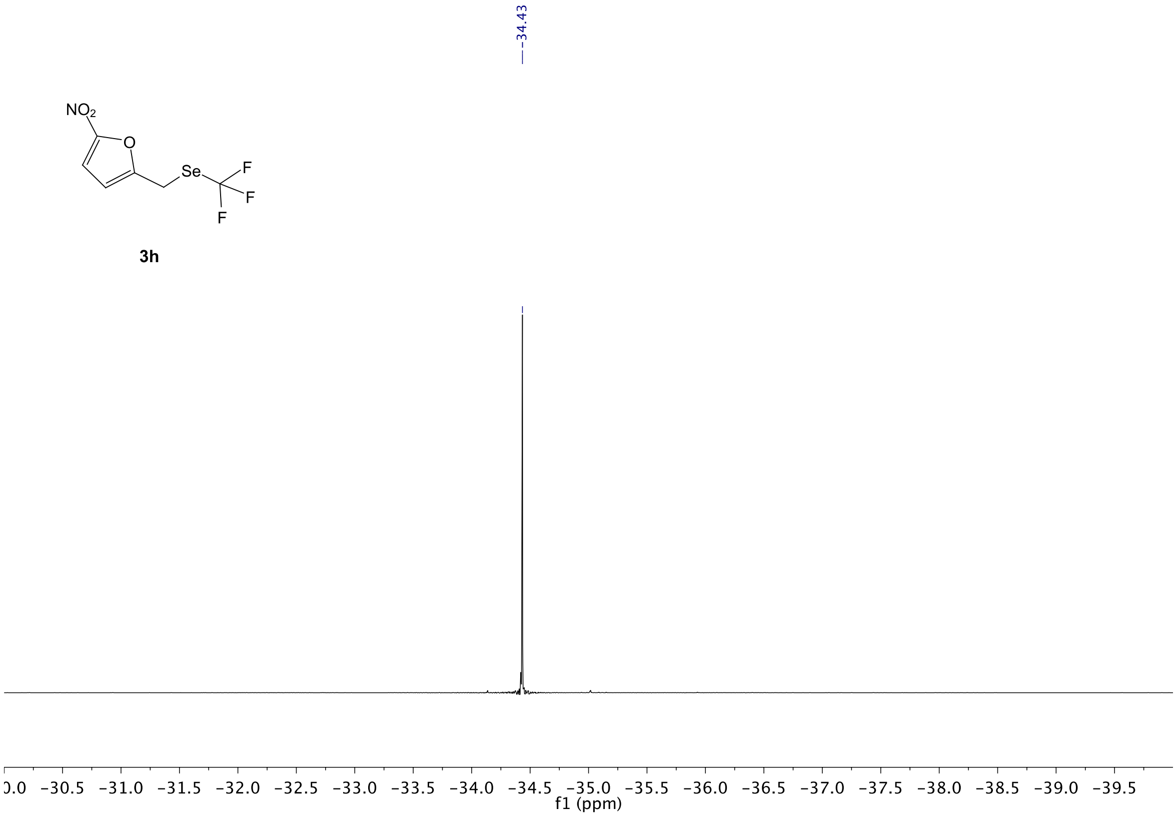

-34.43
NO2
O
Se
F
F
F
-30.5 -31.0 -31.5 -32.0 -32.5 -33.0 -33.5 -34.0 -34.5 -35.0 -35.5 -36.0 -36.5 -37.0 -37.5 -38.0 -38.5 -39.0 -39.5
f1 (ppm)

3h

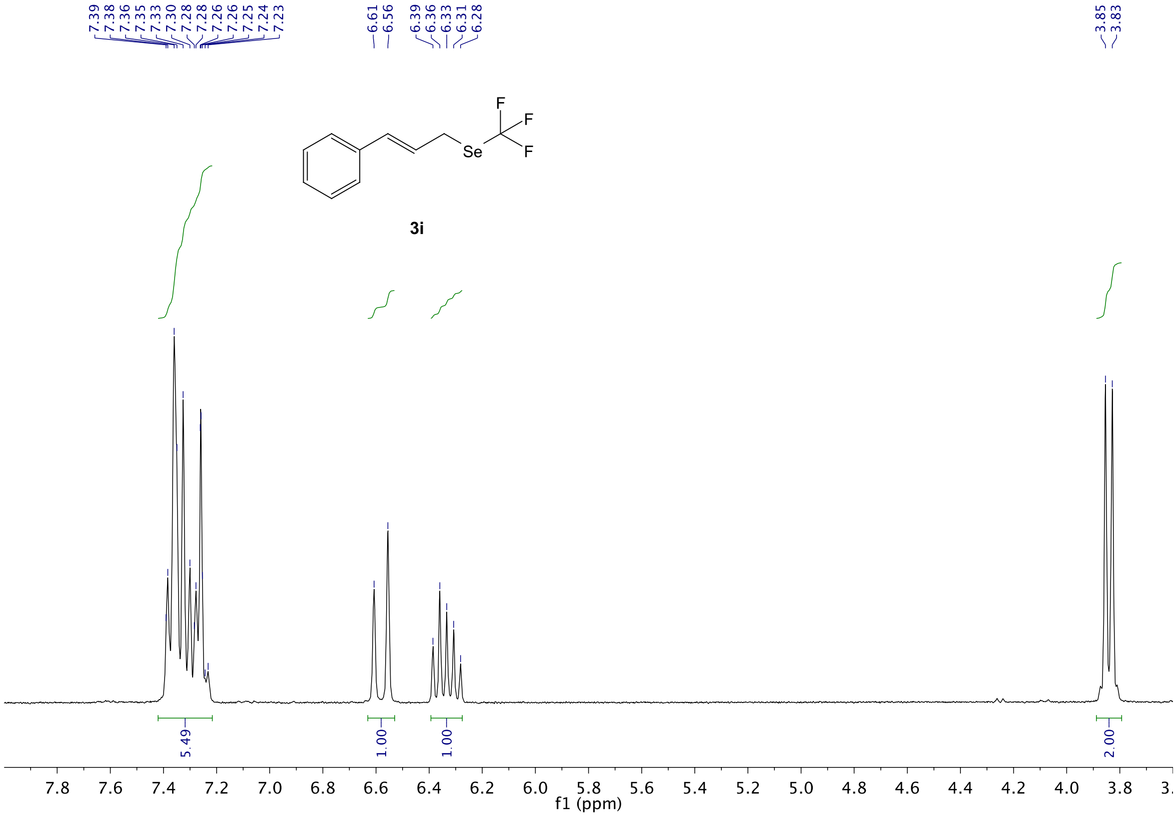
7.39 7.38 7.36 7.35 7.33 7.30 7.28 7.28 7.26 7.26 7.25 7.24 7.23
6.61 6.56
6.39 6.36 6.33 6.31 6.28
3.85 3.83
F F F Se
3i
5.49
1.00
1.00
2.00
7.8 7.6 7.4 7.2 7.0 6.8 6.6 6.4 6.2 6.0 5.8 5.6 5.4 5.2 5.0 4.8 4.6 4.4 4.2 4.0 3.8
f1 (ppm)

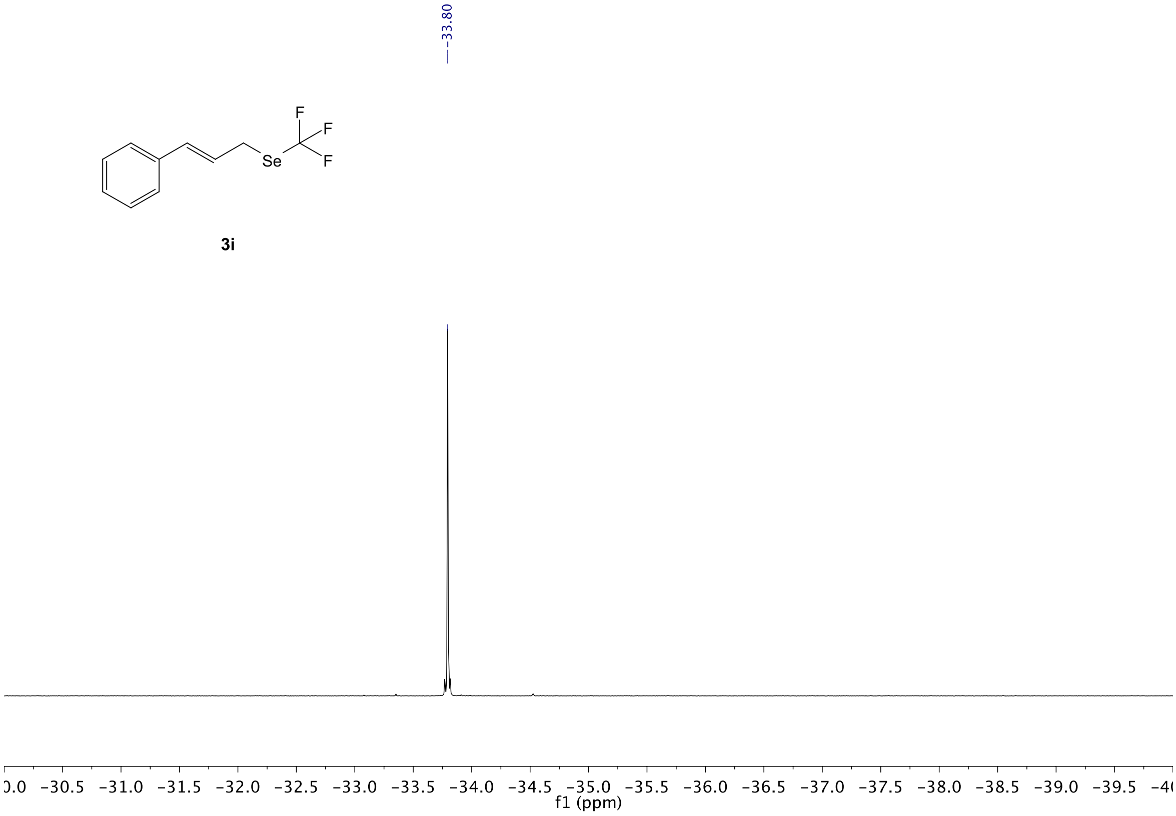
-33.80
F F F Se
3i
-30.5 -31.0 -31.5 -32.0 -32.5 -33.0 -33.5 -34.0 -34.5 -35.0 -35.5 -36.0 -36.5 -37.0 -37.5 -38.0 -38.5 -39.0 -39.5
f1 (ppm)

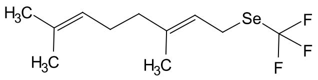

3j

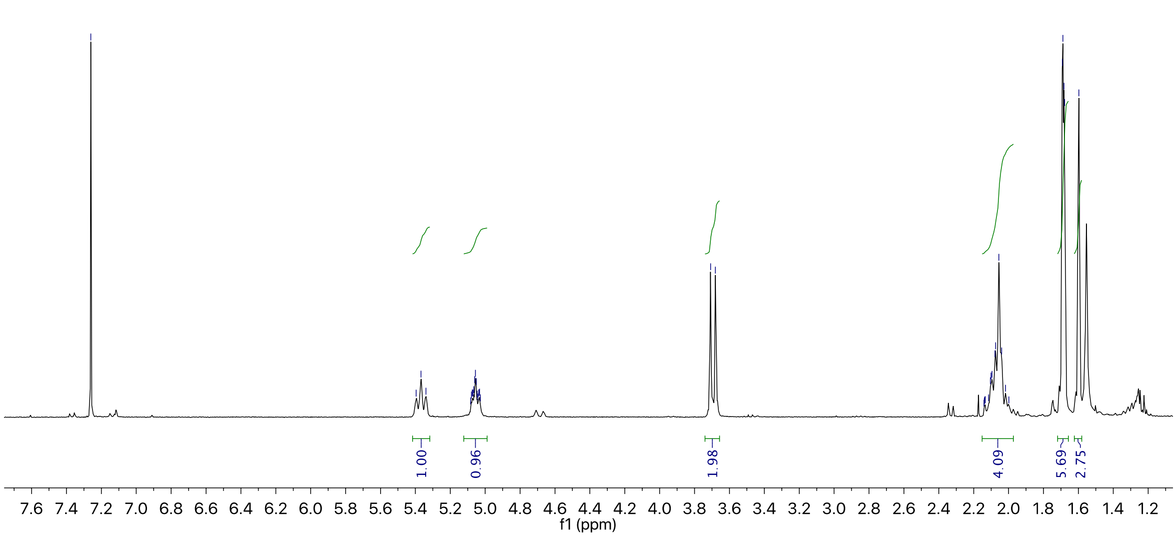

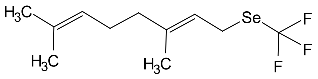

3j

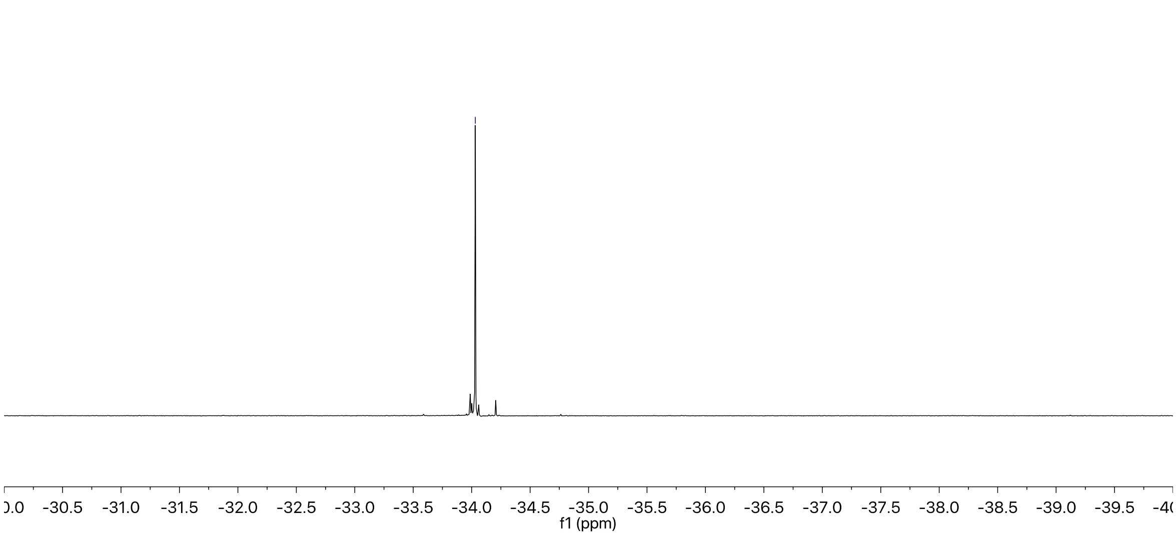

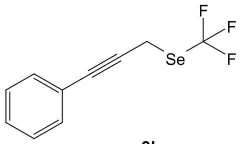
F
F
F
Se

3k

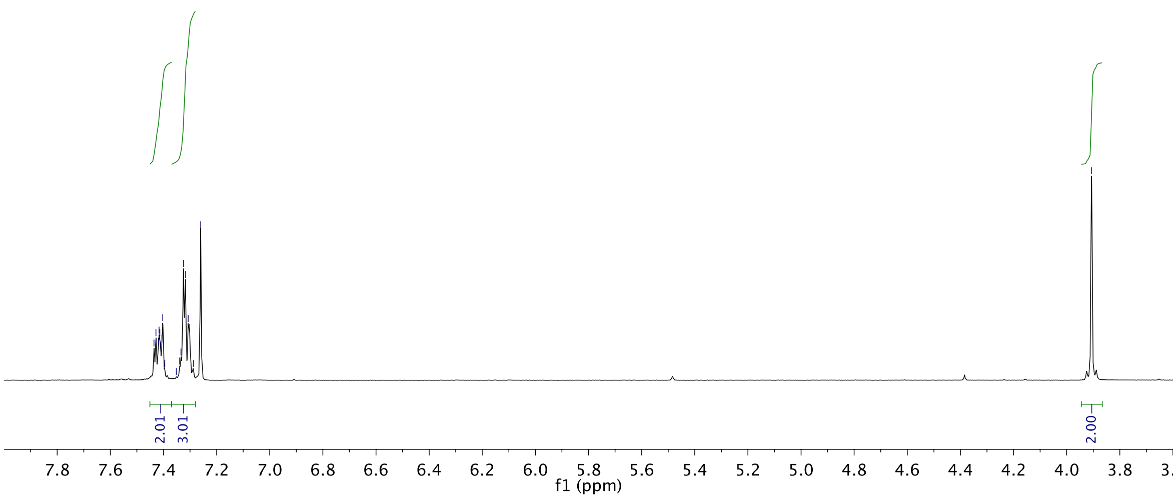
7.44
7.43
7.42
7.42
7.41
7.41
7.40
7.39
7.35
7.34
7.33
7.32
7.32
7.31
7.30
7.29
7.26
3.91
2.01
3.01
2.00
7.8 7.6 7.4 7.2 7.0 6.8 6.6 6.4 6.2 6.0 5.8 5.6 5.4 5.2 5.0 4.8 4.6 4.4 4.2 4.0 3.8
f1 (ppm)

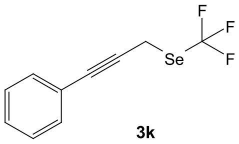
F
F
F
Se

3k

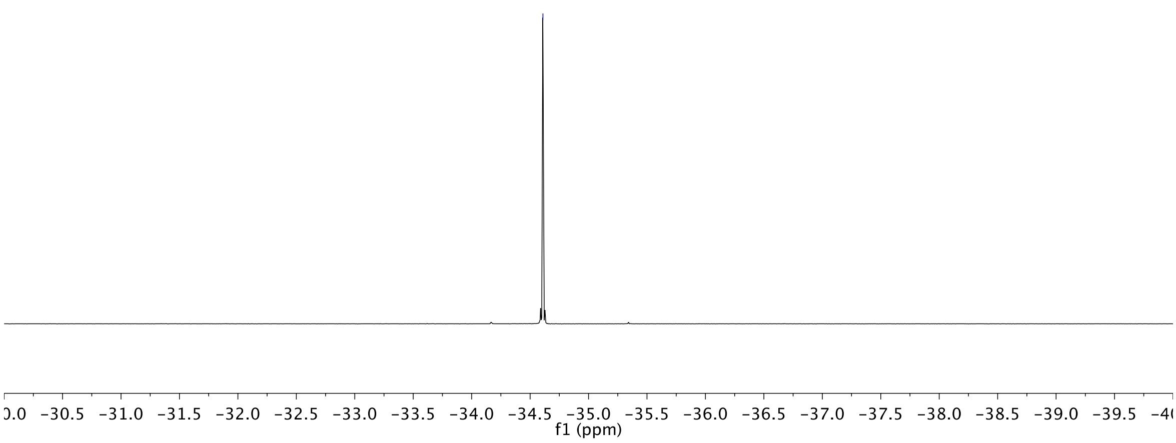
-34.61
-30.5 -31.0 -31.5 -32.0 -32.5 -33.0 -33.5 -34.0 -34.5 -35.0 -35.5 -36.0 -36.5 -37.0 -37.5 -38.0 -38.5 -39.0 -39.5
f1 (ppm)

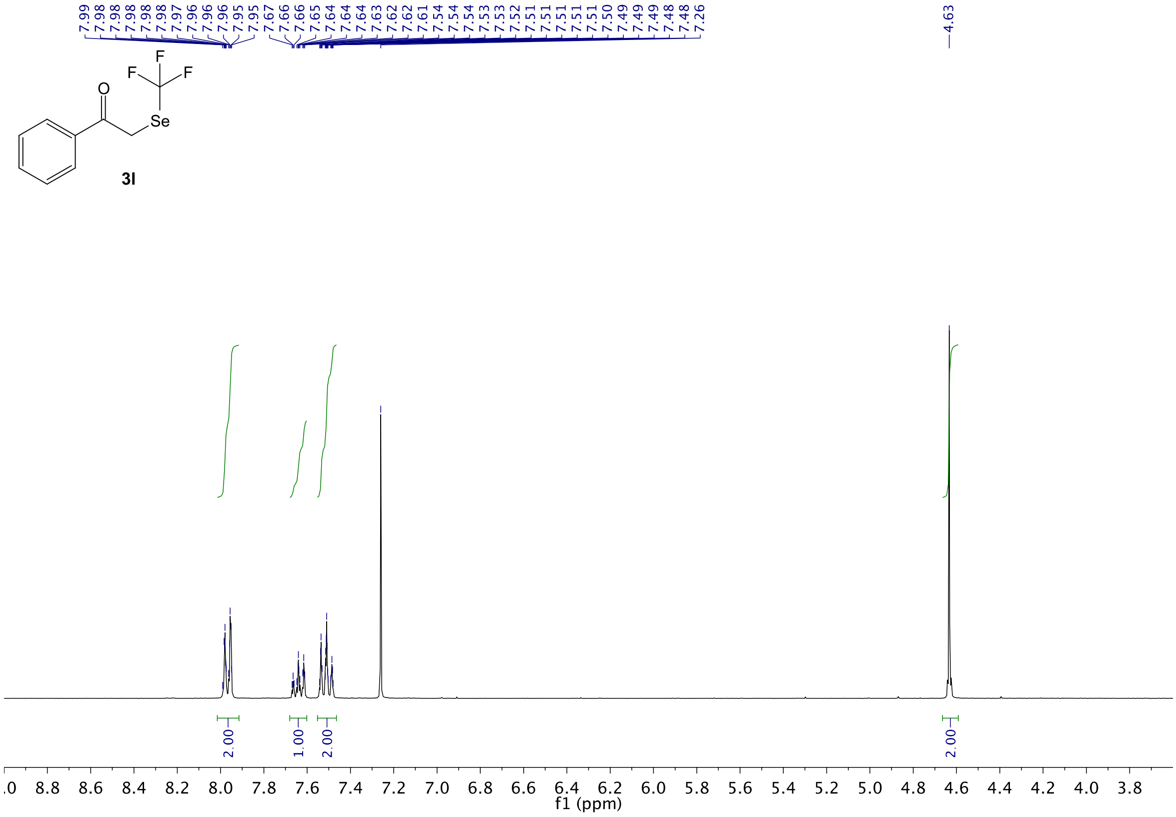

7.99 7.98 7.98 7.98 7.98 7.97 7.96 7.96 7.96 7.95 7.95 7.67 7.66 7.66 7.65 7.64 7.64 7.64 7.63 7.62 7.61 7.54 7.54 7.54 7.53 7.53 7.52 7.51 7.51 7.51 7.51 7.50 7.49 7.49 7.48 7.48 7.26
4.63
F
F
F
O
Se
3l
2.00
1.00
2.00
2.00
.0 8.8 8.6 8.4 8.2 8.0 7.8 7.6 7.4 7.2 7.0 6.8 6.6 6.4 6.2 6.0 5.8 5.6 5.4 5.2 5.0 4.8 4.6 4.4 4.2 4.0 3.8
f1 (ppm)

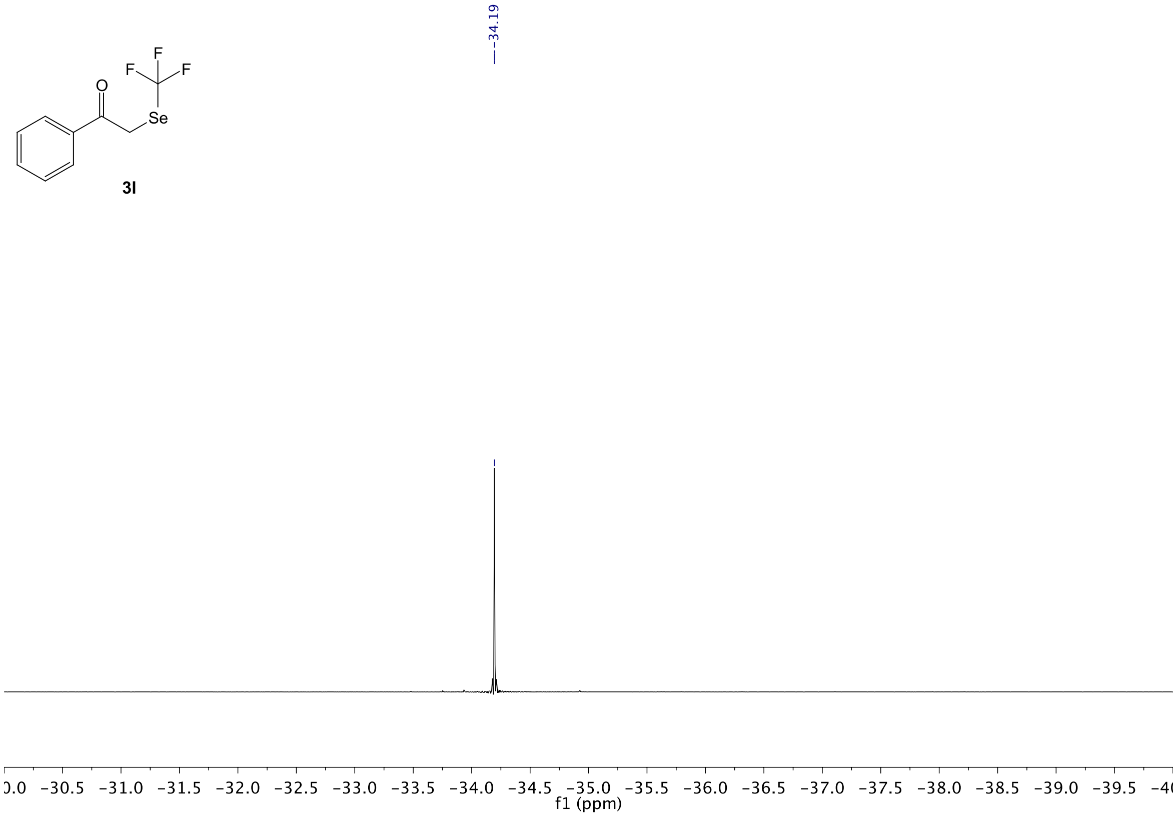

-34.19
F
F
F
O
Se
3l
).0 -30.5 -31.0 -31.5 -32.0 -32.5 -33.0 -33.5 -34.0 -34.5 -35.0 -35.5 -36.0 -36.5 -37.0 -37.5 -38.0 -38.5 -39.0 -39.5 -4(
f1 (ppm)

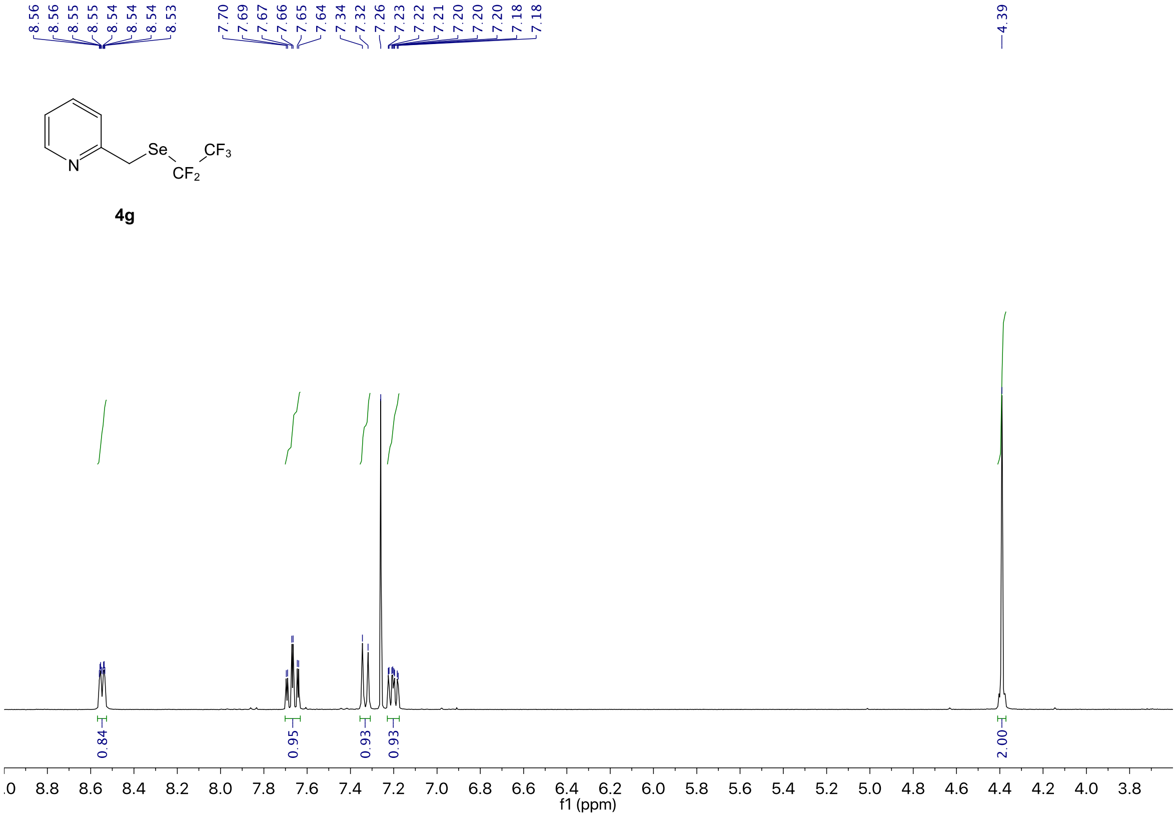

Se
CF3
CF2
N
4g
f1 (ppm)

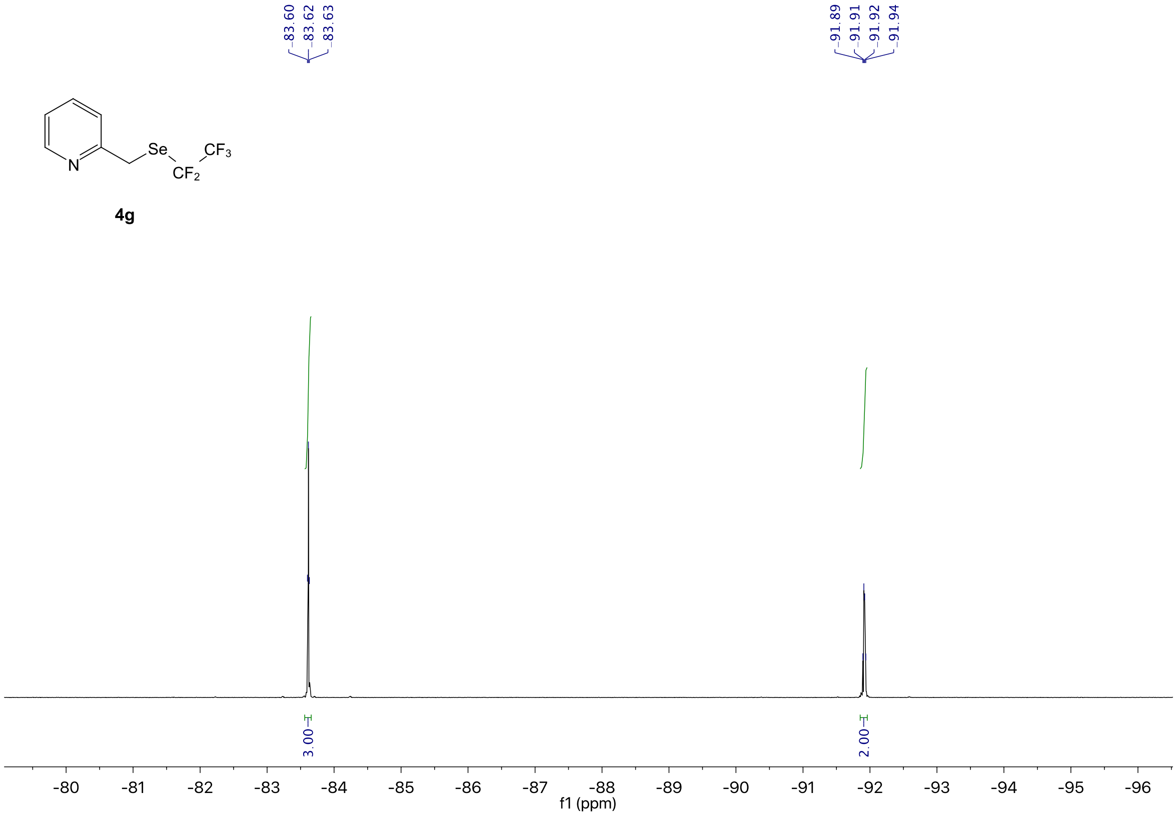

Se
CF3
CF2
N
4g
f1 (ppm)

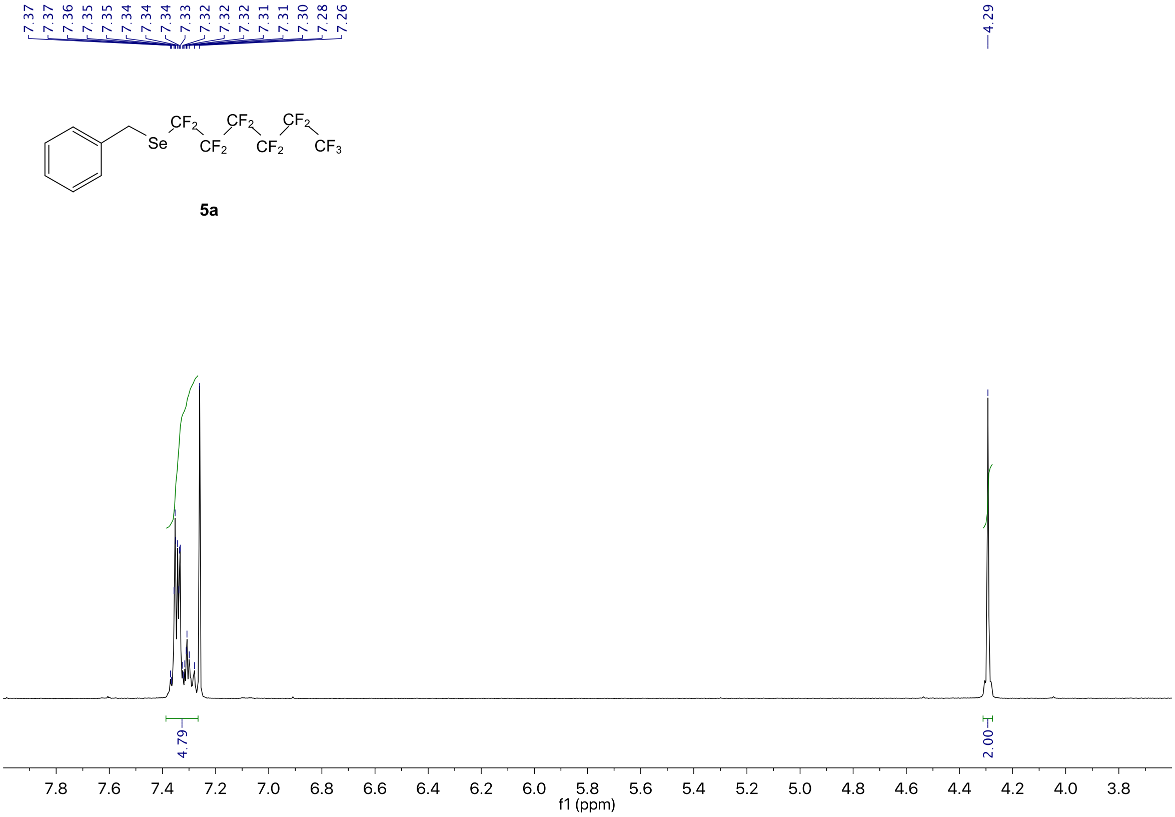
Se
CF_2
CF_3
5a
4.79
2.00
f1 (ppm)

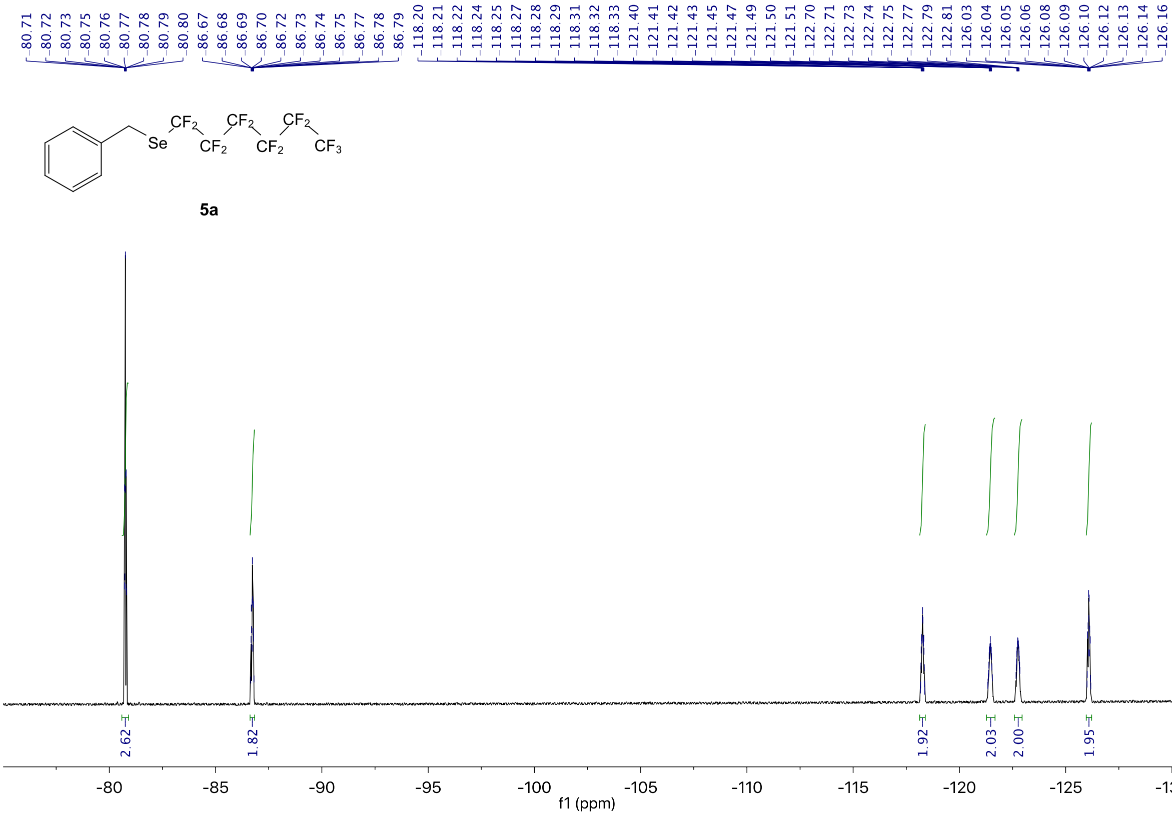
Se
CF_2
CF_3
5a
2.62
1.82
1.92
2.03
2.00
1.95
f1 (ppm)
